# How fungi defend themselves against microbial competitors and animal predators

**DOI:** 10.1371/journal.ppat.1007184

**Published:** 2018-09-06

**Authors:** Markus Künzler

**Affiliations:** Eidgenössische Technische Hochschule Zürich, Department of Biology, Institute of Microbiology, Zürich, Switzerland; Geisel School of Medicine at Dartmouth, UNITED STATES

## Lifestyle exposes filamentous fungi to antagonists

Filamentous fungi arrange their cells in linear, coenocytic arrays, referred to as hyphae, that extend at their tips and are able to branch and fuse, leading to a loose, three-dimensional network referred to as mycelium [1]. This architecture represents an optimal adaptation to the osmotrophic lifestyle of fungi in that it maximizes the surface for nutrient absorption and enables the fungus to efficiently reach and colonize its substrates. Some hyphae of the long-lived and constantly renewed vegetative mycelium may differentiate in other, more compact tissues, e.g., the (usually) short-lived and spore-producing fruiting bodies formed by dikaryotic fungi during their sexual reproduction. The different fungal tissues are exposed to different types of antagonists dependent on the ecological niche of the fungus. The vegetative mycelium of a saprophytic fungus, e.g., is exposed to other microorganisms that compete for the same nutrients and may feed on the degradation products released by the action of the hydrolytic enzymes secreted by the fungus. Accordingly, nutrient-rich substrates, such as the dung of herbivores, are battlefields of competing saprophytic bacteria and fungi [2]. On the other hand, the lack of motility and high content of nutrients make both the fungal vegetative mycelium and the fruiting bodies attractive dietary resources for animal predators. Accordingly, soil-inhabiting fungi are an important dietary resource for soil arthropods and nematodes [3].

## The main defense strategy of fungi is chemical defense

**Fig 1:**
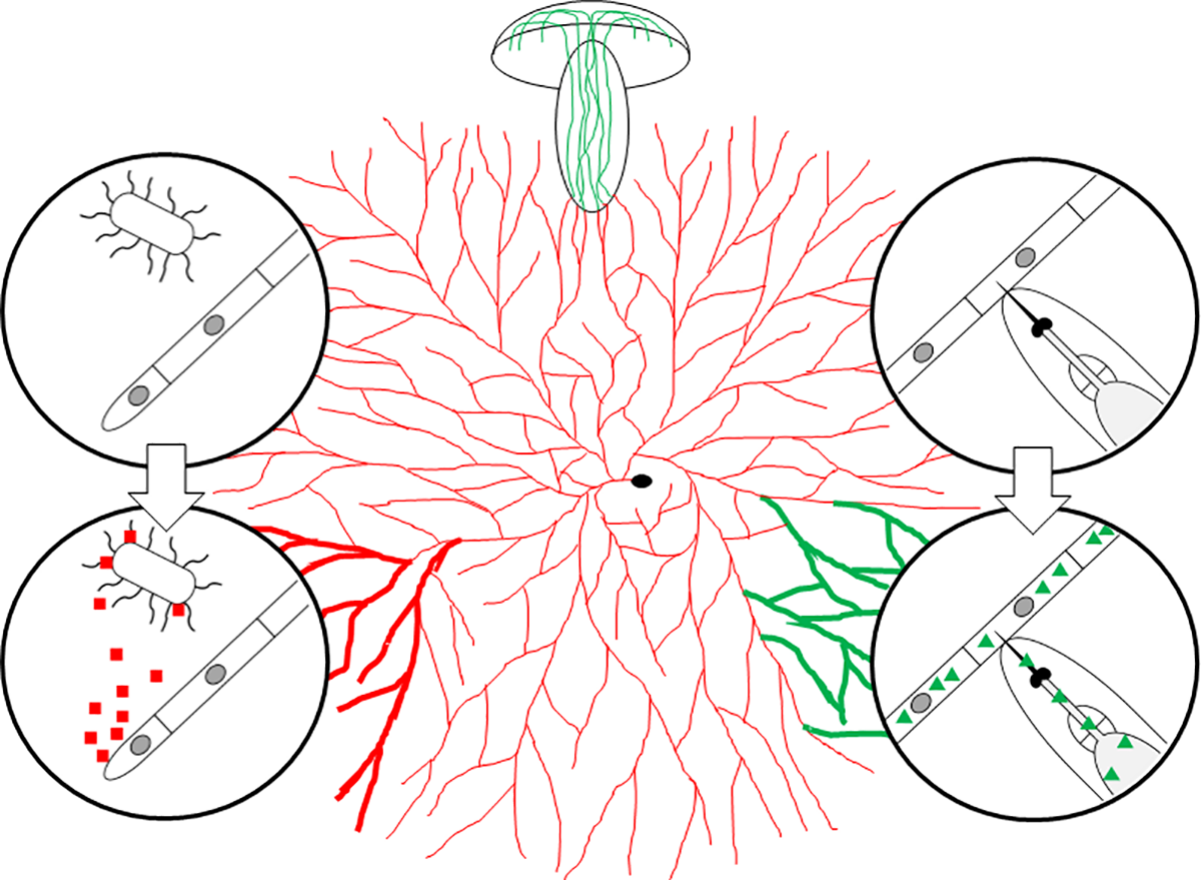
Regulation of the chemical defense of filamentous fungi (on the example of a mushroom) against microbial competitors and animal predators, exemplified by bacteria and fungivorous nematodes (adapted from Fig 1 in [9]). The fungus is represented by its vegetative mycelial network originating from a spore (black oval) and a fruiting body (mushroom) arising from that network. The circles show close ups on the competition between the fungal hyphae and bacteria (left) and predation by fungivorous nematodes (right) and the induction of respective fungal defense effectors; fungal nuclei are represented by grey ovals, extracellular antibacterial defense effectors by red squares, and intracellular defense effectors against nematodes by green triangles. Specific examples of antibacterial and antinematode effectors and their properties are listed in Table 1. Fungal hyphae producing the two types of defense effectors are colored respectively. Autonomous and antagonist-dependent production of defense effectors is indicated by thin and thick hyphae, respectively. The indicated spatial restriction of antagonist-dependent defense effector production in the fungal mycelium is hypothetical.

Fungi have evolved different strategies to increase their competitiveness for nutrient acquisition toward other microorganisms and to protect themselves from predation by animals. Similar to plants, the main defense strategy of fungi is chemical defense, i.e., the production of toxins impairing the growth, development, or viability of the antagonists by the fungus [4]. These defense effectors include secondary metabolites [5], peptides (ribosomally or nonribosomally synthesized) [6, 7], and proteins [8] and usually act by binding to specific target molecules of the antagonists (Table 1). It has been hypothesized that effectors against microbial competitors are secreted, whereas effectors against metazoan predators are usually stored within the fungal cells and are taken up during predation (Fig 1) [9]. Examples of fungal defense effectors in accordance with this hypothesis are the β-lactam antibiotic penicillin produced by some *Penicillium* species [10], the antifungal lipopeptide pneumocandin B_0_ produced by *Glarea lozoyensis* [11], and the cytotoxic, ribosomally synthesized octapeptide α-amanitin produced by some *Amanita*, *Galerina*, *Conocybe*, and *Lepiota* species [12]. Penicillin is secreted and binds and inhibits extracellular enzymes involved in peptidoglycan biosynthesis, an essential and conserved process in all bacteria [13]. Similarly, pneumocandin B_0_ is secreted and inhibits 1,3-β-D-glucan synthase, one of the main enzymes involved in fungal cell wall biosynthesis and is therefore called “penicillin of the antifungals” [11]. In contrast, α-amanitin is taken up from the fungal cell upon predation and enters epithelial cells of the digestive tract of animal predators where it binds and inactivates the essential and conserved nuclear enzyme RNA polymerase II [14]. Exceptions to the hypothesis are a number of secreted insecticidal and nematicidal secondary metabolites [15]. In addition to the action of toxins, fungi have more subtle ways of chemical defense, e.g., by the production of molecules interfering with bacterial and animal communication. Examples are intracellular lactonases of the coprophilous ink cap mushroom *Coprinopsis cinerea* acting as a sink for quorum sensing signals of gram-negative bacteria [16] and the production of insect juvenile hormones by the mold *Aspergillus nidulans* [17].

**Table 1:** Examples of fungal toxins and their targets.

Toxin	Producing fungus	Regulation of production	Subcellular localization	Target organism	Toxin class	Target molecule	Reference
Gliotoxin	*Aspergillus* spp.	Autonomous	Extracellular	Fungi	Secondary metabolite	Proteasome	[65]
Lovastatin	*Aspergillus terreus*	Autonomous	Extracellular	Fungi	Secondary metabolite	HMG-CoA-reductase	[66]
Strobilurin A	*Oudemansiella mucida*	Fungus-induced	Extracellular	Fungi	Secondary metabolite	Cytochrome b	[24]
Pneumocandin B_0_	*Glarea lozoyensis*	Autonomous	Extracellular	Fungi	Peptide	1,3-β-D-glucan synthase	[11]
Copsin/Plectasin/Micasin	*Coprinopsis cinerea/Pseudoplectania nigrella/Microsporum canis*	Autonomous	Extracellular	Bacteria	Peptide	Lipid II	[22, 67, 68]/[69]/[70]
Penicillin	*Penicillium* spp.	Autonomous	Extracellular	Bacteria	Secondary metabolite	Peptidoglycan transpeptidases	[10]
Enniatin A1 and B1	*Fusarium tricinctum*	Bacterium-induced	Extracellular	Bacteria	Peptide	Membrane (ionophor)	[28]
Aflatoxin B1	*Aspergillus flavus*	Autonomous and damage-induced	Extracellular	Insects	Secondary metabolite	DNA	[29]
α-Amanitin	*Amanita*, *Galerina*, *Conocybe*, and *Lepiota* spp.	Autonomous	Intracellular	Insects/Nematodes	Peptide	RNA polymerase II/III	[12]
Omphalotin A	*Omphalotus olearius*	Autonomous	Extracellular	Insects/Nematodes	Peptide	unknown	[71]
Cyclosporin A	*Tolypocladium inflatum*	Autonomous	Extracellular	Insects/Nematodes	Peptide	Cyclophilin/Calcineurin	[72]
CGL2	*Coprinopsis cinerea*	Autonomous and nematode-induced	Intracellular	Insects/Nematodes	Protein	N-glycoproteins	[23, 73]
MOA	*Marasmius oreades*	Autonomous	Intracellular	Nematodes	Protein	Glycosphingolipids	[74]
Clitocypin	*Clitocybe nebularis*	Autonomous	Intracellular	Insects	Protein	Cysteine proteases	[75]
α-Sarcin	*Aspergillus giganteus*	Autonomous	Intracellular	Insects	Protein	28S rRNA	[76]

**Abbreviations:** CGL2, *Coprinopsis cinereal* Galectin 2; CoA, Coenzyme A; HMG, β-Hydroxy β-methylglutaryl; MOA, *Marasmius oreades agglutinin*.

## Fungal defense can be autonomous and/or antagonist-dependent

The biosynthesis of chemical defense effectors is usually tightly regulated because these molecules are not essential for the viability of an organism, and their biosynthesis requires resources that may be limited [18]. This regulation can be autonomous, i.e., independent of the antagonist and/or antagonist-dependent. Accordingly, it has been shown that the regulation of secondary metabolism and sexual development are coordinated in *A*. *nidulans* [19]; some of the secondary metabolites, whose biosynthesis is restricted to the fruiting body, exert toxicity toward arthropods suggesting that these organs are prey of and therefore require protection from animal predators [20]. Similarly, the concentration of amatoxins of the mushroom *Amanita phalloides*, including α-amanitin, is lowest in the vegetative mycelium and highest in the fruiting body [21]. Analogously, genome-wide gene expression analysis of the vegetative mycelium and young fruiting bodies of the model mushroom *C*. *cinerea* revealed that the secreted antibacterial peptide Copsin is almost exclusively produced in the vegetative mycelium, whereas most of the *C*. *cinerea* genes coding for intracellular insecticidal and nematicidal lectins are specifically expressed in the fruiting body (Fig 1) [22]. This spatiotemporal, autonomous regulation results in an efficient constitutive protection of specific fungal tissues against the most relevant antagonists because some of the defense effectors are already in place when the antagonist attacks the fungus. On the other hand, at least some of the lectin-encoding genes directed against animal predators were induced in the *C*. *cinerea* vegetative mycelium when this tissue was challenged with a fungivorous nematode [23]. Similarly, challenge of the vegetative mycelium of the basidiomycete *Oudemansiella murata* with two different *Penicillium* spp. induced the production of the antifungal strobilurin A [24], and challenge of various ascomycetous molds with bacteria and arthropods led to the induction of various gene clusters coding for the biosynthetic machineries of antimicrobial and cytotoxic secondary metabolites, respectively [25–29]. These results suggest that fungi possess, in addition to an autonomous, tissue-specific defense, also an inducible defense (Fig 1). This type of regulation is also known from the innate defense systems of plants and animals [30].

## Open questions

The presence of innate defense systems in multicellular fungi, plants, and animals suggests that such systems are a universal requirement of multicellular organisms. In order to clarify whether these defense systems are the result of divergent or convergent evolution, the fungal defense system has to be better characterized with regard to three key issues of innate defense.

### What is the plasticity and specificity of the induced chemical defense?

Despite above mentioned reports about the induction of fungal defense effector genes upon challenge with bacterial competitors and animal predators, it is not clear how specific these responses are, since almost no signals, receptors, and signaling pathways responsible for these responses are known. This is in contrast to plants and animals, in which sophisticated and multilayered systems of receptors and signaling pathways responsible for the recognition of antagonist-associated molecular patterns or effectors and the induction of antagonist-specific innate defense responses have been identified [31].

While mere wounding triggers the production of fungal defense effectors in some cases [5, 29], there are a few reports about antagonist-associated molecular patterns perceived by fungi. These patterns include cell wall fragments [32] and quorum-sensing signal molecules [33] in the case of bacteria and nematode developmental signal molecules in the case of animals [34]. Besides these soluble signal molecules or patterns, physical contact between the fungus and the antagonist appears to be required for induction of defense [23, 25]. With regard to pattern recognition receptors, plants and animals use two related sets of receptors, namely Toll-like receptors (TLRs) and nucleotide oligomerization domain (NOD)-like receptors (NLRs) for the extracellular and intracellular perception of signals, respectively [30, 35]. Binding of the molecular patterns by these receptors is often mediated by leucine-rich repeat (LRR) domains. Besides the well-characterized G-protein coupled receptors for endogenous sex pheromones, only a few reports of fungal receptors for specific biotic signals exist. Interestingly, the chemotropic sensing of host plant signals by the plant pathogenic fungus *Fusarium oxysporum* was recently demonstrated to be mediated by the sex pheromone receptor [36], suggesting that these receptors may have a broader specificity. None of the over 600 presently known fungal genome sequences, however, appear to encode TLRs. Hyphal growth of the animal-pathogenic yeast *Candida albicans* was shown to be triggered by direct interaction of bacterial muramyl dipeptides (MDPs) with the LRRs of an intracellular fungal receptor protein containing, in addition, a protein phosphatase and an adenylate cyclase domain [32]. This recognition mechanism is similar to the binding of MDP to the mammalian NLR-type receptor NOD2, which triggers inflammation in response to bacterial infections and whose genetic variation has been implicated in susceptibility to Crohn's disease [37]. NLRs are involved in the hetero-incompatibility reaction between different strains of the same fungal species, and it was hypothesized that these proteins, which are widely spread among fungi [38], might also play a role in the perception of fungal antagonists similar to plants and animals [39]. To our knowledge, there is no experimental evidence for this hypothesis so far.

One of the earliest responses of the plant to herbivory (as well as to pathogen and parasite attack) is the production of reactive oxygen species (ROS) and a rapid increase in intracellular calcium (Ca^2+^) [40]. Analogously, fungi react to biotic and abiotic stress with ROS formation and Ca^2+^ influx into cells, and the formation of ROS is dependent on NADPH-dependent oxidases (Nox) [41]. Since Nox's have also been implicated in fungal differentiation [42], these enzymes may play a dual role in development and defense, as demonstrated for other multicellular organisms. Similar to downstream signaling pathways of plant and animal defense responses [31], above mentioned MDP-receptor in *C*. *albicans* suggests downstream signaling via protein phosphorylation/dephosphorylation and cAMP [32]. Accordingly, mitogen-activated protein kinase high osmolarity glycerol (Hog1p) of the yeast *Saccharomyces cerevisiae* was shown to be phosphorylated in response to bacterial lipopolysaccharide (LPS) [43]. In terms of transcription factors involved in the response of fungi to antagonists, to our knowledge, only one example has been reported so far. Overexpression of the transcription factor remediation of secondary metabolism (RsmA) in *A*. *nidulans* lead to induction of transcription factor Aflatoxin biosynthesis regulator (AflR), which in turn resulted in increased expression of an antipredation secondary metabolite gene cluster and avoidance of the transformed mycelium by *Folsomia candida* [44]. In addition, the velvet family of fungal regulators, involved in the above-mentioned coordination of secondary metabolism and sexual development in the same fungus, contains a DNA-binding domain that is structurally related to the main transcription factor NF-κB at the end of the animal TLR-signaling cascade [45].

### Is there a systemic defense response and priming?

In plants, the induction of plant defense effector proteins is not restricted to sites of herbivory but can be propagated to other parts of the same plant or even neighboring plants that have not yet been in contact with the antagonist [46, 47]. This propagation of the defense response relies on amplification of the originally perceived signal by the generation of endogenous signal molecules and the transmission of these molecules within the plant and even to other plants. Endogenous signal molecules implicated in local and systemic plant defense are some plant hormones (salicylic acid, abscisic acid, ethylene) [48], plant oxylipins [49], green leaf volatiles (GLVs) [50], peptides [51], and above-mentioned ROSs [52]. In addition to this chemical signal transmission, this systemic defense response of plants may also be mediated by membrane depolarization [53]. Signaling within fungal mycelia has been studied in some ascomycetes, and a variety of volatile and nonvolatile endogenous signal molecules have been identified [54]. As examples, conidiation of the ascomycetous mold *Trichoderma* is dependent on the 1-octen-3-ol [55], a volatile compound that is also produced by basidiomycetes [56]; oxylipins, which are known endogenous signal molecules in plants and animals, are also known as endogenous signal molecules modulating sexual development and the host interaction of pathogenic fungi [57]. Little is known, however, about the role of such molecules in fungal defense. Interestingly, two genes coding for fatty acid oxygenases involved in the biosynthesis of oxylipins in *A*. *nidulans* are induced upon grazing of the mycelium by larvae of the fruitfly *Drosophila melanogaster* [58], suggesting a possible dual function of these signal molecules in development and defense, as suggested for mosses [59]. The spatial distribution and the propagation of the induced defense response within a fungal mycelium have not been studied so far.

The original dogma that innate immune systems are not able to build up an immunological memory has recently been reconsidered both for plants and animals, and epigenetic histone modifications have been implicated in this process [47, 60]. To our knowledge, the only reports about persistence of an up-regulated defense response in mycelia in the absence of the antagonist, a phenomenon referred to in plants and animals as priming, are two studies by Rohlfs and coworkers [26, 58]. In these studies, the authors show that grazing by *D*. *melanogaster* larvae and the soil arthropod *F*. *candida* induces resistance of the mycelium toward grazing by these predators even after a period of 6 hours without grazing. These results suggest that the induction of the chemical defense in fungi is preserved for some time to protect the mycelium from further damage by predation. There is no data on the spatial distribution of this defense response in the mycelium, however. Interestingly, the induction is dependent on the master regulator of secondary metabolism, the LaeA-velvet system known to act via histone methylation [19], suggesting an epigenetic mechanism for defense gene induction and priming. Accordingly, induction of defense-related metabolic gene clusters in *A*. *nidulans* by bacteria was shown to involve histone acetylation [61].

### What is the ecological significance of fungal defense?

Only a few studies, amongst the two above-mentioned studies of *A*. *nidulans* [26, 58], have addressed the ecological significance of fungal defense in terms of fungal resistance toward grazing. Previous to these studies, it had been shown that *A*. *nidulans* mutants lacking the master regulator of secondary metabolism LaeA and transformants overexpressing the transcription factor RsmA are more susceptible to grazing by *D*. *melanogaster* larvae and more resistant to grazing by *F*. *candida*, respectively [44, 62]. Accordingly, aflatoxin production correlated with the fitness of different *Aspergillus flavus* isolates with regard to grazing by *D*. *melanogaster* larvae [29].

## Relevance and impact

Future in-depth characterization of the fungal innate defense against microbial competitors and animal predators is not only important in terms of basic research, e.g., the evolution of innate defense in eukaryotes, but also in terms of applied research. Fungi are a rich source of chemically diverse natural products, many of which are used for antagonistic interactions. These compounds have a high potential to be used as drugs in management of relevant human or animal diseases and pests. As many of these compounds are produced only in response to an antagonist, investigations of fungal antagonistic interactions are key to exploitation of this “treasure of nature” [63, 64].
